# Exploring Ciprofol Alternatives: A Comprehensive Review of Intravenous Anesthesia Options

**DOI:** 10.7759/cureus.57581

**Published:** 2024-04-04

**Authors:** Nandha kumar Durai Samy, Karuna Taksande

**Affiliations:** 1 Anaesthesiology, Jawaharlal Nehru Medical College, Datta Meghe Institute of Higher Education & Research, Wardha, IND

**Keywords:** future research, safety profile, clinical efficacy, pharmacology, ciprofol alternatives, intravenous anesthesia

## Abstract

Ciprofol is a recently developed, short-acting γ-aminobutyric acid receptor agonist sedative that is more potent than propofol. Still, there have been few clinical studies of this agent to date. This review explores alternative intravenous anesthesia options to ciprofol, considering their pharmacology, clinical efficacy, safety profile, and practical considerations. While ciprofol offers advantages such as rapid onset and predictable offset, concerns regarding its safety profile and individual variability in response have prompted the search for alternatives. Propofol, etomidate, ketamine, and dexmedetomidine are discussed as established options, each with unique characteristics and potential benefits. Emerging agents, including remimazolam, sufentanil, alfaxalone, and brexanolone, are examined for their potential role in anesthesia management. Recommendations for future research include large-scale comparative studies, optimization of dosing strategies, and personalized approaches guided by pharmacogenomic insights. Ultimately, the future of intravenous anesthesia lies in a multifaceted approach that integrates evidence-based practices, technological innovations, and individualized patient care to enhance safety, efficacy, and patient satisfaction across the perioperative continuum. Collaboration among stakeholders will be crucial in advancing the field and shaping the future landscape of intravenous anesthesia options.

## Introduction and background

Ciprofol, also known as cyclopropyl-methoxycarbonyl etomidate, is a relatively new intravenous anesthetic agent that has gained attention for its potential advantages in anesthesia induction and maintenance. As an alternative to traditional agents like propofol and etomidate, ciprofol offers a unique pharmacological profile characterized by rapid onset, predictable offset, and minimal cardiovascular effects. Its mechanism of action involves potentiation of γ-aminobutyric acid type A (GABAA) receptors, leading to central nervous system depression and anesthesia induction [1]. In clinical practice, ciprofol has been utilized in various surgical procedures, including but not limited to general surgery, orthopedic surgery, and endoscopic procedures. Its favorable pharmacokinetic properties make it particularly suitable for situations requiring rapid anesthesia induction and short-duration procedures [2].

Despite its promising characteristics, several factors drive the need to explore alternatives to ciprofol. Firstly, while ciprofol offers advantages in certain aspects, it may only be suitable for some patients or surgical settings due to individual variability in response and specific clinical considerations. Additionally, concerns regarding its safety profile, including potential neuroexcitatory effects and drug interactions, have prompted clinicians to seek alternative options that may offer comparable efficacy with improved safety profiles [3]. Furthermore, the emergence of drug shortages and supply chain disruptions has underscored the importance of diversifying anesthesia options to ensure continuity of care and mitigate risks associated with reliance on a single agent [4].

This review aims to provide a comprehensive examination of alternative intravenous anesthesia options to ciprofol. By critically evaluating the pharmacology, clinical efficacy, safety profile, and practical considerations of these alternatives, this review aims to assist clinicians in making informed decisions regarding anesthesia management. Additionally, this review seeks to identify gaps in current knowledge and areas for further research to optimize anesthesia practice and patient outcomes.

## Review

Pharmacology and mechanism of action

Overview of Ciprofol’s Pharmacokinetics

Ciprofol, a novel alternative to propofol in clinical intravenous anesthesia, is a short-acting intravenous sedative known for its high efficacy, excellent selectivity, and minimal adverse reactions. Its pharmacokinetic properties closely resemble those of propofol, and it boasts approximately four to five times greater potency as a highly selective GABAA receptor potentiator [1,5]. Upon intravenous administration, ciprofol swiftly disperses throughout the tissues and undergoes rapid elimination, with concentrations in specific tissues surpassing those in plasma. Its primary metabolic pathways include oxidation, glucuronic acid binding, and sulfuric acid metabolism, with the primary metabolites excreted via urine and feces [1]. Phase II clinical trials have demonstrated the pharmacokinetic equivalence of ciprofol to propofol in the induction and maintenance of general anesthesia for elective surgical procedures [1]. Regulatory approval has been granted for ciprofol’s use in inducing and maintaining general anesthesia and for providing sedation in intensive care settings [6]. Furthermore, a comprehensive drug-drug interaction study has explored the impact of sodium divalproex on ciprofol in terms of pharmacokinetics and pharmacodynamics [7].

Mechanism of Action

Ciprofol, a novel alternative to propofol in clinical intravenous anesthesia, operates primarily by enhancing the ion channel’s activity mediated by GABAA receptors [1,5,6]. It functions as a positive allosteric modulator and direct agonist of the GABAA receptor, exhibiting a higher receptor affinity for ciprofol than propofol. Incorporating a cyclopropyl group into the core structure of ciprofol enhances its steric and stereoselective effects, resulting in greater anesthetic potency relative to propofol [1]. Studies have indicated that ciprofol is approximately four to six times more potent than other phenol derivatives like propofol or fospropofol [8]. As a result, the pharmacological characteristics of ciprofol position it as a promising option for various anesthesia and sedation applications.

Pharmacodynamics

Ciprofol stands as a novel sedative-hypnotic medication authorized for the induction and maintenance of general anesthesia, as well as for sedation in intensive care settings. Its pharmacological attributes and mode of action have undergone extensive scrutiny through numerous studies. Functioning as a highly selective agonist of the GABAA receptor akin to propofol, ciprofol exhibits a potency approximately four to five times greater than propofol. At a study dose of 0.4 mg/kg, it displays favorable pharmacokinetic traits, pharmacodynamic responses, and safety profiles. Comparative analyses have revealed that ciprofol presents a more consistent hemodynamic profile and a reduced frequency of adverse events compared to propofol. Moreover, its therapeutic index surpasses that of propofol by 1.5 times, suggesting potential for simplified clinical use, particularly among elderly patients. Nevertheless, further investigations are imperative to establish its enduring safety and efficacy, particularly when juxtaposed with propofol across diverse clinical contexts, as well as to discern potential drug-drug interactions [1,5-7,9].

Clinical efficacy and safety of ciprofol

Efficacy in Different Surgical Settings

Ciprofol has demonstrated efficacy and safety across diverse surgical settings. A systematic review and meta-analysis of clinical studies revealed that ciprofol exhibited comparable safety and tolerability to propofol during both the induction and maintenance of anesthesia [10]. In another study comparing the efficacy and safety of ciprofol to propofol in patients undergoing procedures, ciprofol was found to be as effective as propofol in providing anesthesia for gynecological ambulatory surgery while exhibiting a lower incidence of adverse events [11]. Additionally, ciprofol has shown effectiveness in procedures such as colonoscopy, bronchoscopy, and elective surgeries [10,11]. While ciprofol holds promise, further investigation is imperative to establish its long-term safety and efficacy, particularly in comparison to propofol across various clinical scenarios [1,10].

Safety Profile

Ciprofol, an innovative intravenous anesthetic, has demonstrated efficacy and safety across various clinical contexts. Clinical investigations have shown that ciprofol induces comparable sedation or anesthesia to propofol in nonoperating room environments, boasting high efficacy, excellent selectivity, and fewer adverse reactions [12]. Following intravenous administration, its plasma concentration exhibits a polyphasic decline, resulting in a final elimination half-life of 1.58 to 2.47 hours [10]. Ciprofol’s safety profile, characterized by a stable hemodynamic profile and a reduced incidence of adverse events compared to propofol, positions it as a promising option for clinical intravenous anesthesia [1]. Notably, the drug offers advantages such as improved tolerance, higher sedation satisfaction scores, and a lower occurrence of adverse reactions, particularly in mitigating pain upon injection [1]. Moreover, studies have demonstrated the safety of ciprofol at doses ranging from 0.15 to 0.90 mg/kg in mechanically ventilated patients, with most adverse effects being mild to moderate in severity [13]. Overall, the available evidence underscores ciprofol as a promising alternative to propofol, with a favorable safety profile and considerable potential for clinical application.

Side Effects and Adverse Reactions

**Figure 1:**
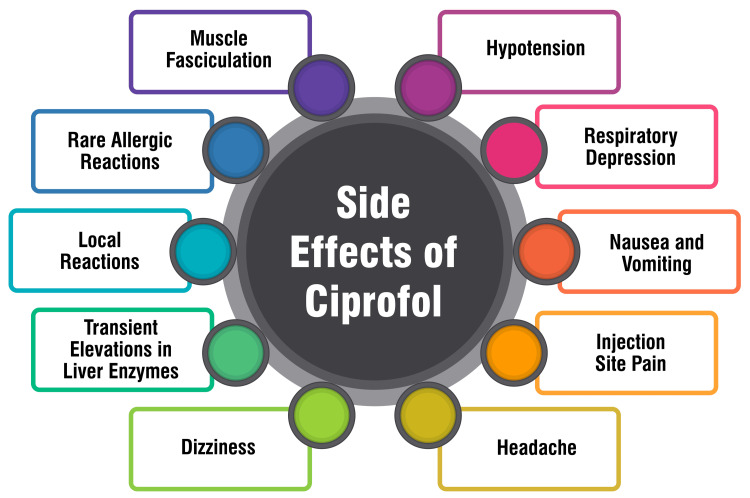
Side effects of ciprofol This figure was self-created by the corresponding author.

Ciprofol stands out for its high efficacy, favorable selectivity, and reduced incidence of adverse reactions. It is a promising choice for a spectrum of procedures encompassing the induction and maintenance of general anesthesia, sedation during intensive care, and nontracheal intubation surgeries. Numerous clinical studies have underscored its efficacy and safety across diverse settings, ranging from gastroscopy, colonoscopy, and bronchoscopy to elective surgeries. Notably, ciprofol’s pharmacokinetic profile closely resembles that of propofol while boasting a more stable hemodynamic profile and a lower occurrence of adverse events when compared directly to propofol. Although ciprofol may elicit mild to moderate adverse reactions such as muscle fasciculation, hypotension, and respiratory depression, the overall incidence of adverse events remains lower than that observed with propofol. Nonetheless, further research endeavors are warranted to ascertain its long-term safety and efficacy, particularly in comparative analyses with propofol across varied clinical scenarios [1,10,12,13]. The side effects of ciprofol are shown in Figure 1.

Limitations and drawbacks of ciprofol

Pharmacological Limitations

Ongoing studies are underway to investigate potential drug-drug interactions involving ciprofol and other medications, such as esketamine, to ascertain safety and compatibility when administered concurrently [1]. Ciprofol’s pharmacokinetic characteristics, including its distribution in tissues and plasma protein-binding rates, have been subject to investigation. Notably, the drug is swiftly eliminated from most tissues, with concentrations in specific tissues surpassing those observed in plasma. Factors such as age may influence ciprofol’s pharmacokinetics, with the elderly population exhibiting slightly prolonged unconsciousness and shorter recovery times [1]. With an estimated potency of four to five times greater than that of propofol, careful consideration is warranted when determining ciprofol dosage. The disparity in potency necessitates cautious dosage adjustments, as a straightforward mg/kg comparison may not accurately reflect the appropriate administration [14]. While some studies have indicated a lower incidence of adverse reactions associated with ciprofol than propofol, ongoing monitoring and assessment of its safety profile remain imperative. This is particularly crucial when considering special populations and specific clinical scenarios [1,14]. Ciprofol has shown a decreased risk of injection pain in comparison to propofol, a common adverse reaction associated with the latter [14]. Despite its promise as an alternative to propofol, continued research is indispensable to comprehensively comprehend its pharmacological constraints and guarantee its safe and efficacious application in clinical practice, particularly when juxtaposed with existing anesthesia options.

Clinical Challenges

**Figure 2:**
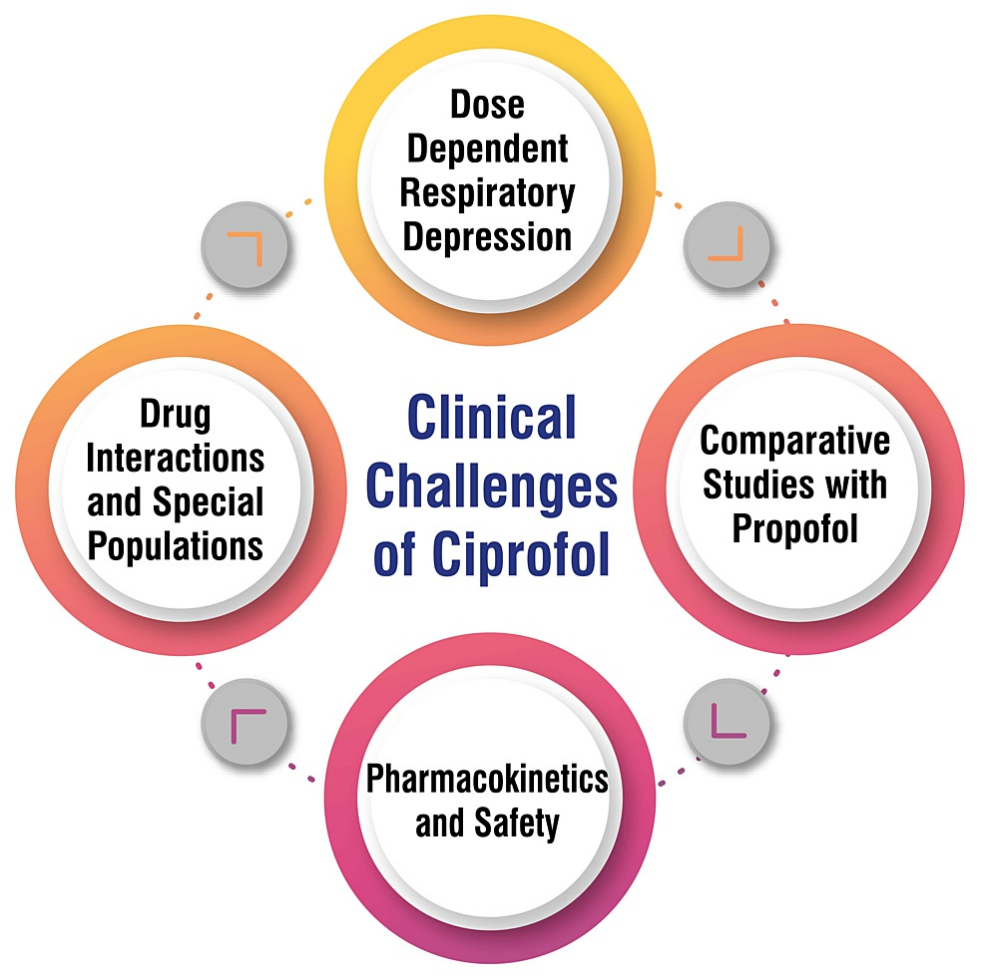
Clinical challenges of ciprofol This figure was self-created by the corresponding author.

Ciprofol has been linked to dose-dependent respiratory depression, particularly in pediatric and elderly patients, as well as individuals with cardiovascular diseases and hypovolemia [15]. While specific studies have indicated favorable attributes of ciprofol, such as reduced injection pain and respiratory depression, compared to propofol, further comparative research is imperative to determine its superiority or equivalency to propofol across diverse clinical scenarios [15]. An extensive examination of ciprofol’s pharmacokinetics, encompassing parameters like plasma concentration and elimination half-life, has been undertaken. Continually assessing its safety profile remains critical, especially among surgical patients and those undergoing procedural sedation [10]. Ongoing investigations are concentrating on potential drug interactions and the impact of special populations, such as the elderly, on ciprofol’s pharmacokinetics and clinical effects [6]. Despite its potential as an alternative intravenous anesthetic, addressing the aforementioned challenges through continued research and clinical evaluation is essential to ensure the safe and effective utilization of ciprofol across various patient demographics and clinical environments. The clinical challenges of ciprofol are shown in Figure 2.

Economic Considerations

Ciprofol, a novel alternative to propofol in intravenous anesthesia, presents notable pharmacological advantages, boasting a wide therapeutic window and high efficacy [16]. However, considering the limitations and drawbacks associated with ciprofol, particularly in economic considerations, is imperative. While clinical trials have underscored the safety and efficacy of ciprofol, its current price stands approximately three times higher than that of propofol. Although broader utilization is anticipated to decrease the price of ciprofol, a comprehensive analysis of its economic implications relative to other anesthetics remains lacking [10]. Furthermore, the long-term economic ramifications of ciprofol, encompassing factors such as resource utilization and cost-effectiveness, await comprehensive evaluation. Hence, further research endeavors are warranted to thoroughly assess the economic aspects of ciprofol as an intravenous anesthetic option [10].

Alternative intravenous anesthesia options

Etomidate

Pharmacology and mechanism of action: Etomidate is a short-acting intravenous anesthetic agent utilized for inducing general anesthesia, facilitating tracheal intubation, cardioversion, and other procedures necessitating brief anesthesia periods. Its mechanism of action involves binding to the GABAA receptor, thereby instigating the opening of chloride channels and inducing dose-dependent cortical depression [17,18]. Renowned for its rapid onset of action, brief duration of effect, and favorable hemodynamic profile, etomidate is particularly suitable for patients experiencing hemodynamic instability. Metabolized by hepatic and plasma esterases, etomidate exhibits a biexponential decline in plasma concentration. Notable side effects include venous pain upon injection and myoclonus, while an induction dose may lead to adrenocortical suppression lasting four to six hours [17,19]. A unique toxicity associated with etomidate involves its capacity to inhibit adrenal steroid synthesis, posing potential challenges for patients with significant cardiovascular ailments or those necessitating prolonged sedation [20].

Clinical efficacy and safety: Etomidate, recognized as a short-acting intravenous hypnotic, finds application in inducing general anesthesia, facilitating rapid sequence intubation, and addressing other scenarios where brief anesthesia is essential. Its utility extends to procedural sedation, maintenance of anesthesia, and short operative procedures such as reducing dislocated joints, tracheal intubation, cardioversion, dilation, curettage, or cervical conization [17]. As a GABAA receptor agonist, etomidate is an anesthesia induction agent, distinguished by its advantageous hemodynamic and respiratory properties, as it does not precipitate cardiovascular or respiratory depression [21]. A significant advantage of etomidate is its minimal impact on the cardiovascular system. It is a preferred choice for patients with hemodynamic instability, cardiac disease, or even those in hemorrhagic shock [21,22]. However, etomidate’s propensity to suppress the adrenal axis precludes its suitability for prolonged infusions [21]. Notable side effects associated with etomidate encompass postoperative nausea and vomiting, pain upon injection, and myoclonic movements or involuntary muscle contractions during induction and emergence [21]. In the pediatric population, etomidate is considered safe as an induction agent [21].

Comparisons with ciprofol: Etomidate, a short-acting intravenous hypnotic, has garnered approval for various anesthesia applications, including induction of general anesthesia, rapid sequence intubation, and other short-term anesthesia needs. Its versatility extends to procedural sedation, maintenance of anesthesia, and short operative procedures such as reducing dislocated joints, tracheal intubation, and cardioversion [17]. Notably, etomidate exhibits a favorable hemodynamic profile upon induction, characterized by minimal blood pressure depression, rendering it particularly suitable for patients with significant cardiovascular disease [17]. Renowned for its rapid onset of action, brief duration of effect, and rapid metabolism, etomidate is less prone to causing a notable drop in blood pressure than other induction agents, making it a preferred choice for hemodynamically unstable individuals [17,23]. In contrast, ciprofol emerges as a novel alternative to propofol for intravenous anesthesia, offering distinct advantages such as enhanced tolerance, heightened sedation satisfaction scores, and a reduced incidence of adverse reactions, particularly in mitigating pain upon injection [22]. With a potency approximately four to five times higher than propofol, ciprofol necessitates a lower dosage to achieve sedation compared to propofol [17]. Both etomidate and ciprofol find utility in procedural sedation and anesthesia induction. Etomidate is recognized for its favorable hemodynamic profile and the swift onset of action, while ciprofol exhibits advantages in tolerance, sedation satisfaction, and reduced adverse reactions. Ultimately, the choice between etomidate and ciprofol hinges on specific clinical requirements, patient characteristics, and the preferences of healthcare providers. Each agent offers unique benefits, and the selection should be tailored to meet the patient’s needs and the clinical scenario’s demands.

Ketamine

Pharmacology and mechanism of action: Ketamine, a dissociative anesthetic, has been utilized for over five decades as a safe anesthetic agent with potent analgesic properties [24]. Its mechanism of action is intricate, involving direct interaction with the N-methyl-D-aspartate (NMDA) receptor, downstream effects on regulators such as brain-derived neurotrophic factor (BDNF) and mammalian target of rapamycin (mTOR), and impacts of ketamine’s metabolites [25]. Additionally, ketamine enhances the descending inhibitory serotoninergic pathway and exhibits antidepressant effects [24,25]. Notably, ketamine reverses opioid tolerance and generally preserves normal pharyngeal and laryngeal reflexes while allowing for spontaneous respiration. It enhances or sustains normal skeletal muscle tone and is associated with cardiovascular and respiratory stimulation [26]. Moreover, ketamine has shown effectiveness in treating depression and managing pain at lower subanesthetic doses [25]. Metabolized primarily into norketamine, an active metabolite, ketamine is predominantly eliminated through urine [24].

Clinical efficacy and safety: Ketamine emerges as a versatile medication with a myriad of clinical applications encompassing anesthesia, pain management, and depression treatment. As a dissociative anesthetic, it induces a trance-like state that offers pain relief, sedation, and amnesia. At subanesthetic doses, ketamine exhibits promise as a rapid and efficacious treatment for depression, with studies showcasing significant improvements in depressive and anxiety symptoms [27,28]. Its mechanism of action involves direct interaction with the NMDA receptor, downstream effects on regulators such as BDNF and mTOR, and the impacts of its metabolites [29]. Predominantly metabolized into norketamine, an active metabolite, ketamine’s pharmacokinetics and pharmacodynamics have been extensively studied, affirming its status as a safe and efficacious anesthetic drug with potent analgesic properties [24]. Notably, ketamine boasts a rapid onset of action and a robust impact on depression, rendering it a valuable option for individuals grappling with treatment-resistant depression [27]. Moreover, investigations have revealed that a subanesthetic dose of S-ketamine yields superior effects on both pain and depression compared to racemic ketamine in patients with cancer [30]. Overall, ketamine has showcased efficacy and safety across a spectrum of clinical applications, with its potential for depression treatment particularly salient.

Comparisons with ciprofol: Ciprofol emerges as a novel intravenous anesthetic agent developed as an alternative to propofol. Notably, it has demonstrated effectiveness and favorable tolerability, boasting a potency approximately four to five times higher than propofol. This higher potency translates to a reduced requirement of active drugs for inducing sedation compared to propofol [31,32]. Conversely, ketamine serves as a dissociative anesthetic utilized medically for both anesthesia induction and maintenance, alongside its application in depression treatment and pain management [33]. Esketamine, a derivative of ketamine, has been studied as an adjunct to either ciprofol or propofol sedation, aiming to enhance cardiorespiratory stability [32]. Both ketamine and its derivative, esketamine, are recognized for their potent analgesic properties and find utility across a spectrum of anesthetic and analgesic contexts [24]. While ciprofol and ketamine (or its derivative, esketamine) have been investigated for their sedative and anesthetic effects, any specific comparisons in the context of intravenous anesthesia options would necessitate further clinical research and evidence.

Dexmedetomidine

Pharmacology and mechanism of action: Dexmedetomidine stands out as a specific and selective alpha-2 adrenoceptor agonist employed for sedation and analgesia and as an adjunct to anesthesia. Its mechanism of action involves the activation of presynaptic α2-adrenoceptors, which inhibit the release of norepinephrine, thereby diminishing the propagation of pain signals. Furthermore, the activation of postsynaptic α2-adrenoceptors inhibits sympathetic activity, leading to reductions in blood pressure and heart rate. This unique mechanism yields sedation, anxiolysis, and analgesia while minimally affecting respiratory function [34-36]. Studies have unveiled additional facets of dexmedetomidine’s functionality, including its ability to regulate synaptic plasticity and hippocampal long-term potentiation. Moreover, dexmedetomidine has been scrutinized for its potential neuroprotective properties, encompassing antisympathetic effects, apoptosis and oxidative stress inhibition, and attenuation of the inflammatory response [37]. Commercially available as a water-soluble HCl salt and administered intravenously, dexmedetomidine boasts a rapid onset of action and a relatively short half-life, rendering it suitable for diverse clinical applications. These include procedural sedation, augmentation of local analgesia techniques, and utilization in ICUs and perioperative settings [35]. Despite its merits, dexmedetomidine’s use warrants vigilant monitoring due to potential side effects such as bradycardia and hypotension [37]. Nonetheless, its unique pharmacological profile and potential for organ protection render it a valuable option for sedation and analgesia across various clinical scenarios.

Clinical efficacy and safety: Dexmedetomidine, an α2-adrenoceptor agonist, is valued for its sedative, anxiolytic, and analgesic attributes. Extensively studied for its clinical efficacy and safety, it finds application in procedural sedation, mechanical ventilation, and critical care contexts. Dexmedetomidine has effectively diminished the necessity for rescue medications like propofol during infusion, shortened mechanical ventilation duration, and exhibited favorable sedative and analgesic effects with minimal respiratory depression [38-40]. Its distinctive pharmacodynamic profile, characterized by sedative and analgesic-sparing effects, renders it invaluable across diverse clinical scenarios. Notably, dexmedetomidine exerts a minimal impact on respiratory function but may induce transient hypertension, bradycardia, and hypotension due to its peripheral vasoconstrictive and sympatholytic properties [35]. Commercially available as a water-soluble HCl salt and administered intravenously, dexmedetomidine holds promise across various applications. However, further research is warranted to explore its potential off-label indications. These include its use in pediatric and geriatric populations as an adjunct to prolong peripheral or spinal nerve blocks and its capacity to curtail opioid consumption [35].

Comparisons with ciprofol: Dexmedetomidine, an intravenous sedative, has undergone extensive study regarding its efficacy and safety in procedural sedation and anesthesia. Research findings have indicated that dexmedetomidine effectively met the primary efficacy endpoint, demonstrating a significantly higher percentage of patients not requiring rescue propofol during infusion than a placebo group. Notable adverse events associated with dexmedetomidine include hypotension, respiratory depression, and bradycardia. Furthermore, comparisons with other sedatives like midazolam and propofol have revealed potential benefits, including reducing mechanical ventilation time [39,40]. In contrast, ciprofol emerges as another intravenous anesthetic agent subject to scrutiny regarding its efficacy and safety, particularly within the ICU setting. Research has indicated that ciprofol has a shorter half-life than dexmedetomidine and has demonstrated promising sedation and delirium control outcomes in ICU patients. It is anticipated to exhibit comparable efficacy to propofol while presenting fewer complications in ICU patients [41,42]. Both dexmedetomidine and ciprofol have served as focal points in clinical trials evaluating their efficacy and safety in sedation and anesthesia, each displaying potential advantages in various clinical scenarios. Further research endeavors and comparative studies are warranted to delineate each intravenous anesthetic agent’s specific roles and benefits in clinical practice.

Emerging intravenous anesthesia agents

Remimazolam

Remimazolam emerges as a burgeoning intravenous anesthetic agent, showing considerable promise in clinical applications. Classified as a short-acting benzodiazepine, it boasts a rapid onset and offset of action, rendering it suitable for both general anesthesia and procedural sedation. Clinical trials have underscored its efficacy and safety in facilitating anesthesia and sedation across diverse procedures, with specific studies drawing comparisons to other commonly utilized agents such as propofol and midazolam [43-45]. The pharmacological profile of remimazolam is marked by its rapid metabolism into an inactive metabolite, contributing to its predictable duration of action and favorable hemodynamic and respiratory effects compared to alternatives like propofol [45]. Its controllable short-acting properties position it as a potentially valuable asset in anesthesia. Remimazolam has garnered approval for use in various anesthesia and sedation contexts in multiple countries [43,44]. Increasingly acknowledged as a safe and effective alternative to widely used sedatives for intravenous anesthesia, remimazolam’s unique pharmacological profile makes it a promising option in clinical practice.

Sufentanil

Sufentanil emerges as a burgeoning intravenous anesthetic agent utilized for inducing and maintaining anesthesia, serving as an analgesic during labor and delivery, and managing severe acute pain. Functioning as an opioid analgesic, it is administered through intravenous, epidural, and sublingual routes. Sufentanil primarily exerts its pharmacologic effects on the central nervous system, offering analgesic and sedative properties. Notably, it is renowned for its remarkable potency, with specific studies suggesting it to be up to 10 times more potent than fentanyl. Sufentanil is currently under evaluation for various applications, and its distinctive properties position it as a promising option for intravenous anesthesia [46-48].

Alfaxalone

Alfaxalone stands as an intravenous anesthetic agent, recognized as an analog of progesterone and a synthetic neuroactive steroid. With a longstanding history of use in veterinary medicine for inducing and maintaining general anesthesia, a novel formulation of alfaxalone featuring preservatives has recently entered the market in the United States. This formulation boasts a shelf life of 28 days post-initial use. Approved for intravenous administration to induce and sustain general anesthesia in dogs and cats, alfaxalone also finds off-label use via the intramuscular route for induction and sedation. Its attributes include a swift onset of action, the provision of satisfactory muscle relaxation, and the absence of accumulation post-repeat dosing. Compatible with several sedative and analgesic agents, alfaxalone presents advantages such as enhanced tolerance, heightened sedation satisfaction scores, and reduced adverse reactions, particularly in effectively mitigating injection pain. Despite its merits, alfaxalone usage may entail potential risks, such as respiratory depression and apnea, while lacking analgesic properties. Notably, recovery from alfaxalone anesthesia sans supplementary drugs may be tumultuous, potentially leading to manifestations like paddling, vocalization, rigidity, and myoclonus in dogs and paddling and trembling in cats. As an emerging intravenous anesthetic agent, alfaxalone holds promise for clinical application [48-51].

Brexanolone

Brexanolone is a novel intravenous anesthesia agent categorized as a neuroactive steroid gamma. It comprises an aqueous formulation of the steroid allopregnanolone, a metabolite of progesterone generated by the brain, corpus luteum, and placenta. Notably, brexanolone has garnered FDA approval specifically for postpartum depression. However, its accessibility is currently restricted to a registry due to its association with somnolence and excessive sedation, as denoted by a black box warning. Beyond its application in postpartum depression, brexanolone has undergone investigation as an adjunctive therapy in cases of super-refractory status epilepticus (SRSE). Findings indicate tolerability among SRSE patients with diverse etiologies, demonstrating a high success rate in weaning off temporary life-sustaining measures (TLAs) before tapering brexanolone. The formulation comprises an isotonic solution containing 5 mg/ml of allopregnanolone buffered in 250 mg/ml sulfobutylether-B. Dosing protocols for brexanolone are weight based, with infusion adhering to recommended schedules [52-54].

Considerations for clinical practice

Patient-Specific Factors

Patient-specific factors play a pivotal role in determining appropriate drug dosing and treatment strategies. The most prevalent factors influencing dosing are age, weight, kidney function, and liver function [55]. However, it is crucial to also consider additional categories and unique patient factors necessitating dosage adjustments, including comorbidities, medication history, and special patient populations like pediatrics and geriatrics [55]. While clinical practice guidelines offer a foundation for treatment recommendations, they must be tailored to accommodate individual patient values and preferences [56]. When formulating treatment plans, healthcare providers should be attentive to nonspecific factors, such as patient preferences, history, values, and individual differences [56-58]. The American Society of Anesthesiologists’ Physical Status Classification System provides a practical framework for quantifying perioperative risk based on patient-specific risk factors identified during physical assessments [58]. In essence, personalized treatment recommendations should encompass a comprehensive array of patient-specific factors to ensure the delivery of safe and effective care.

Surgical Setting and Requirements

The efficacy and safety of ciprofol, a novel intravenous anesthetic, have undergone extensive evaluation in various clinical studies, positioning it as a potential alternative to existing intravenous anesthetic agents. Critical considerations for its application in the surgical milieu encompass dosing, efficacy, safety, and profiles of adverse reactions. Ciprofol has demonstrated effectiveness for both procedural sedation and anesthesia induction in surgical patients, boasting high success rates and exhibiting a lower incidence of adverse events than propofol [8-10,12,42]. In a multicenter phase 2a clinical trial, participants aged 18-65, nonpregnant, and nonlactating with a BMI of 19-30 kg/m^2^ were recruited. The trial aimed to assess the success rates of anesthesia induction following initial bolus doses of ciprofol, with results indicating notably high success rates [9]. Furthermore, a separate study focused on the induction of general anesthesia in gynecological surgery patients revealed that ciprofol was comparable to propofol in terms of induction success rates, with the added benefits of a reduced incidence of injection site pain and more stable bispectral index changes [8]. Detailed guidelines regarding the dosing and administration of ciprofol in the surgical setting have been outlined in these studies, encompassing considerations for initial bolus doses, maintenance doses, and the potential necessity for top-up doses. Moreover, investigations into the safety and efficacy of ciprofol for sedation of mechanically ventilated patients in ICUs have yielded crucial insights into its utility in this critical care setting [42].

## Conclusions

In this review, we have explored a range of intravenous anesthesia alternatives to ciprofol, highlighting propofol for its reliability and comparable pharmacokinetics and etomidate for its hemodynamic stability despite concerns regarding adrenal effects. We also touched upon unique options like ketamine and dexmedetomidine, as well as the potential of newer agents such as remimazolam and alfaxalone, emphasizing the need for further study to establish their roles fully. The path forward involves comprehensive research, tailored dosing regimens, and a personalized medicine approach, underpinning a future where advanced, evidence-based anesthesia practices enhance patient outcomes and satisfaction in the evolving landscape of surgical care.
